# Associations of Urban and Green Land Covers and Heat Waves in 49 U.S. Cities between 1992 and 2019

**DOI:** 10.3390/ijerph19137688

**Published:** 2022-06-23

**Authors:** Sakib Hasan, Woonsup Choi, Sangjun Kang

**Affiliations:** 1 Department of Geography, University of Wisconsin-Milwaukee, Milwaukee, WI 53211, USA; hasan6@uwm.edu (S.H.); choiw@uwm.edu (W.C.); 2 Department of Urban Planning & Real Estate, Gangneung-Wonju National University, Gangneung 25457, Korea

**Keywords:** urban expansion intensity index, green expansion intensity index, Spearman correlation, heat wave

## Abstract

The study aimed to examine how changing land use conditions are related to the occurrence of heat waves. The employed methods were (1) the Urban Expansion Intensity Index (UEII) and the Green Expansion Intensity (GEII) for 49 cities in the U.S. between 1992 and 2019; (2) Spearman correlation analyses of heat wave indicators including frequency, season, duration, and intensity for UEII, and GEII, respectively. Major findings include the following: (1) urban areas have increased rapidly with an average UEII value of 1.5; (2) green Areas have increased at a slow pace, which have a GEII average value of 0.017, where the median value is −0.1, meaning the green area is declining in most U.S. cities; (3) The UEII and heat wave duration show a negative relationship with a significant correlation ($\gamma_{s}$ = −0.296 and ρ = 0.04); (4) UEII and heat wave intensity show a positive relationship with a significant correlation ($\gamma_{s}$ = 0.32 and ρ = 0.027). It was found that heat wave intensity can be a public health issue in high urban expansion intensity areas. The results imply that cities would be better in a more compact pattern with more expanded green areas to mitigate the negative health impacts of heat waves on citizens in urban areas. It is noticeable that there are some patterns to be investigated further in the context of urban developments and heat wave characteristics.

## 1. Introduction

A heat wave is an important weather event affecting both humans and other organisms and one of the leading causes of weather-related deaths [1]. Kim and Kang [2] argue that the deterioration of the thermal environment due to high temperatures might affect the health of urban dwellers. Heatwaves enhance the dangers of heat exposure. In the U.S., thousands of people seek medical attention each year after being exposed to excessive heat [3]. The projected demographic and climatic trends suggest that heat stress is likely to remain an important health concern in the U.S. [4]. Some European counties are approaching the high-temperature problem caused by heatwaves from a national security point of view [5]. Heatwaves cause not only heat-related diseases or death for humans but also the death of livestock or wildlife and this impact is exacerbated by high humidity, which slows the pace at which perspiration evaporates from the skin [5,6]. There is a multitude of definitions of heatwave or heat stress. A heatwave, with various quantitative definitions, is generally considered to be a period of extreme and unusual warmth [7]. On the other hand, heat stress is quantitatively differently defined in different studies and appears to have implications for human health [8,9,10]. However, regardless of each different quantitative definition, there is no difference reported in that heat-related deaths occur when the body’s ability to cool itself through increased blood circulation and perspiration is outstripped by the environment’s rapid rise in temperature. It is reported that heat stress affects the elderly, the young, and persons with mental diseases or chronic illnesses the most [11].

The occurrence of heat waves is known to be affected by not only the climate but also urban land uses. Urban areas are places where a large number of people and property are concentrated. Thus, urban dwellers are particularly sensitive to heat waves due to the urban heat island effect [12]. Choi [13] explored the nighttime heat stress in two Midwestern regions in the U.S., including the cities of Minneapolis and Milwaukee, U.S. He reported that heat stress is projected to increase both in frequency and duration and the urban heat island effect. The urban thermal environment has a relationship with urban development patterns [14,15]. The relationship between specific development types and heatwaves provides a very important opportunity for better understanding the relationship between land use and heatwaves. Compact cities have characteristics such as high-density development, centralized and clustered development, mixed land use, and secured green space [16,17,18]. For the relationship between compacted urban spatial structure and heatwaves, Stone and Rodgers [19] used a sprawl index developed from a mixed land use concept including centrality, continuity, and density for 53 large cities in the U.S., in order to determine the relationship between the number of days of heatwaves. They proposed that in a city with a relatively compact urban spatial form, heat waves occur on fewer days. On the other hand, Newman [20] suggests that sufficient green space is not secured in a compact city with a high population density in a small area. Furthermore, he argues that an increase in urban density contributes to traffic energy consumption by reducing vehicle use, whereas an increase in density worsens air pollution concentration by concentrating air pollution sources.

The energy use linked with land use is said to have a strong relationship with the urban heatwave phenomenon. Previous research indicates that energy usage is related to land use. The increased energy use has a negative effect on the urban thermal environment. The thermal environment benefits from a compact urban spatial layout, which is primarily due to the reduction of pavement area on the ground surface and the reduction in energy consumption due to vehicle traffic [14,21]. Kim and Kang [2] also presented the result that a high average population density of the entire city leads to an increase in population, which in turn worsens the urban thermal environment. However, it is well known that urban expansions with low-density developments are decreasing the number of open spaces and fragmenting natural habitats, which can have a significant ecological impact. The open space loss in 274 metropolitan areas between 1990 and 2000 was 1.4 million ha [22]. The loss of green space creates a detrimental impact on psychological health as urban green spaces can help with mental health issues. They were found to buffer stress or the risk of depression across the United States [23].

Green spaces in cities are thought to be an effective approach to mitigate urban heat island effects and bring comfort to residents [24]. An increase in green space can certainly impact the cooling effect. One study in Kumamoto City, Japan [25] showed the coefficients of association between the green area ratio and air temperature as being negative, with daytime values of −0.678 and nighttime values of −0.753. The urban green space cooling effect describes the ability of urban green spaces to influence the surrounding area in addition to cooling the actual space. The most significant factors to consider when it comes to the cooling impacts of urban green areas are the intensity and density of the cooling, both of which can help urban designers and planners deal with urban heat islands [24].

Recently, green areas are discussed in the context of urban development land use patterns as a form of green infrastructure. It is represented as a form of natural or artificial networks of ecological systems at all spatial scales, including urban and regional areas, with the enhanced quality and quantity of green spaces, their multifunction, and the interconnection between habitats [26,27,28]. Green infrastructure can preserve the natural ecosystem’s values and functions [29,30]. It also provides various potential benefits that are not only limited to people but which also affect the natural ecosystem, including biodiversity, maintenance of natural landscape processes, and stormwater and flood management [31,32]. Green infrastructure is also understood as a means to reduce urban air pollution. Kim et al. [33] researched the analysis of the effect of fine dust reduction and green infrastructure. They suggested the expansion of the green infrastructure to ensure better air quality in urbanized areas. In recent years, the application of the green infrastructure concept is highly recommended in the establishment and application of urban development plans for the reduction of air pollution concentration in Korea [34].

This study aimed to examine how changing land use conditions is related to the occurrence of heat waves. Previous studies did not investigate the correlation between the change in an urban area and urban green space and the characteristics of heatwaves. This study, particularly, tried to find the correlation between the urban and green expansion intensity index (UEII and GEII) and heatwave frequency, duration, season, and intensity. The UEII method is widely used to determine the intensity and direction of urban growth. A study in Tripoli, a metropolitan area in Libya, used UEII to conclude that the urban sprawl was high in urban fringe areas rather than in the regions nearer to the CBD [35]. UEII is also significant for determining a stepwise linear regression to evaluate the driving forces of urban expansion. Xuzhou city in China increased by 67% between 1987–1994 and the total population growth was considered the primary factor for the urban expansion along with the other independent variables such as agricultural production, infrastructure investment, gross domestic product, and the proportion of primary, secondary, and tertiary industries in GDP [36]. The expansion pattern or direction can be easily identified through the spatial and temporal distribution of local Moran’s I and UEII. Zhuhai city in China evolved from a double center city into a multi-center city and the urban areas were aggregated around Xiangzhou and Doumen districts from 1990 to 2015 [37]. Another study in the Democratic People’s Republic of Korea used the combination of UEII and Local Moran’s I in the ten largest cities in Democratic People’s Republic of Korea and established that the urban expansion was visible in the western inland and eastern coastal regions [38]. This study expands the use of UEII and GEII by correlating them with the urban thermal environment. To better understand the urban thermal environment, including heatwaves, it is necessary to understand the occurrence of heat wave indicators in the context of land use conditions, including urban land use patterns and sizes. Sustainable land-use planning aims to meet various social demands from the environmental, societal, and economical aspects with multi-constraints [39]. This study will enhance our understanding of sustainable land use planning through the exploration of heatwaves and land uses. These efforts provide further research questions regarding the mechanisms of urban thermal phenomena and the associated public health impacts in urban areas.

## 2. Materials and Methods

**Figure 1:**
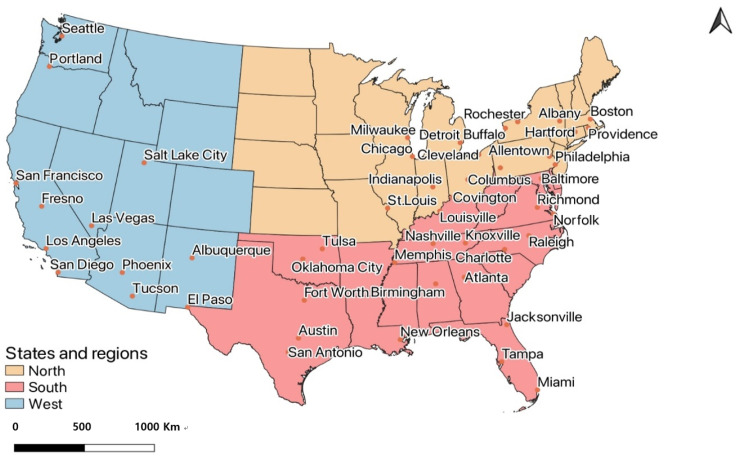
Location of 49 cities in the study area.

This paper collected data for urban and green areas and heatwave occurrence. The heatwave data includes distinct characteristics of heatwaves, such as their frequency, duration, season, and intensity. Then, the expansion of the urban and green area for each year using the Urban Expansion Intensity Index (UEII) and Green Expansion Intensity Index (GEII) was calculated. Finally, we tried to identify a significant correlation between urban and green areas and the characteristics of heatwaves using Spearman correlations to better understand what property in a heatwave is changing with the change of land use. The land cover data were collected from multiple sources. The 1992 land cover data were collected from the U.S. Department of Energy website. The United States Geological Survey (USGS) National land Cover Data (NLCD) has one complete raster dataset for the contiguous United States. The data do not cover islands such as Puerto Rico or Hawaii. The data were derived from the early to mid-1990s Landsat Thematic Mapper satellite data. The NLCD has 21 classes, each containing distinct land cover classifications. The NLCD for 2019 was compiled from the Multi-Resolution Land Characteristics Consortium (MRLC) website. The NLCD 2019 contained 20 classes, which is fewer than the 1992 NLCD. The classes were simplified using the Anderson Level I class code, which facilitates the comparison of classes for NLCD 1992 and NLCD 2019 [40]. This study used the following classes to declare urban and green areas (Table 1 and Table 2):

The heatwave data were collected from the study of Habeeb et al. [12] where the author collected heatwave data for 50 cities of United States of America. This study tried to have the same cities except for San Juan, the capital of Puerto Rico, because of the data unavailability. The shapefiles for the rest of the 49 cities were accumulated from the United States Census Bureau website (Figure 1). The NLCD of 1992 and 2019 were clipped to the city boundaries through the ArcGIS Pro clip tool. The raster dataset was re-projected to Albers Equal Area Conic Projection as the area of features resembles the area of the earth in this projection system. The area for each class was determined using the calculate geometry tool in square miles for the 49 cities. The area of the cities was checked with the area in the U.S. Census Bureau 2010 data and all city areas were calculated within 5% of these data, which is negligible.

**Figure 2:**
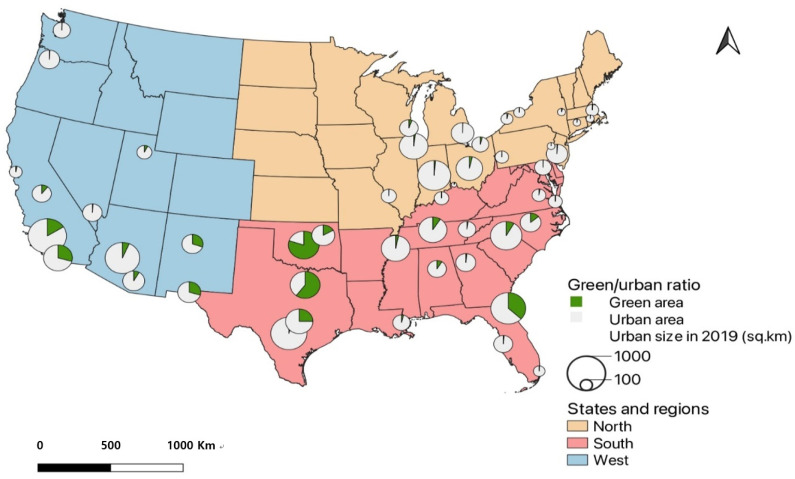
The proportion of urban and green areas in the study area.

UEII and GEII were calculated for the 49 cities using the field calculator tool in ArcGIS Pro. The dataset from Habeeb et al. [12] was added to each city using the join table method containing the heat wave frequency, season, intensity, and duration. As shown in Figure 1, the cities were then grouped into 3 regions: West, South, and North. U.S. Census Bureau divided the U.S. states into 4 regions which are West, Midwest, Northeast, and South [41]. This study aggregated the Northeast and Midwest regions into the North region. Figure 2 presents the proportion of urban and green areas in the 49 cities using the 2019 NLCD. The heat wave data were concatenated using R Studio. The Spearman correlations helped us to visualize the relation between UEII and GEII and heat wave frequency, duration, season, and intensity.

One of the leading causes of weather-related deaths is heat waves [1]. A heat wave, according to the most basic definition, is an extended period of extremely high atmospheric-related heat stress that produces temporary lifestyle changes and may have negative health repercussions for the affected population. An excessive heat event (EHE) is defined by the NCDC (National Climatic Data Center) heat index as any day where the minimum, maximum, or average apparent temperature for each first-order meteorological station in the database exceeds the 85th percentile of the base period (1961–1990) [12]. In this study, a heat wave is defined as any minimum apparent temperature that exceeds the local 85th percentile for two or more days in a row. Apparent temperature is related to both temperature and humidity which was developed by Gaffen and Ross [42]. The equation that is used to determine the apparent temperature is as follows:

A=−1.3 + 0.92T + 2.2e

where A is the apparent temperature (°C), T is ambient air temperature (°C) and e is water vapor pressure (kPa).

We have used four characteristics from the heat wave data for the 49 cities. Heat waves are characterized by frequency, duration, intensity, and length. The number of heat wave events that a city experiences each year is referred to as heat wave frequency. Heat wave duration is the total number of days in a row that a heat wave lasts, and it was averaged annually for each city. The difference between the average heat wave day’s temperature and the local EHE temperature threshold is considered as heat wave intensity. The number of days elapsed from the start of the first heat wave to the end of the last heat wave was counted to determine the length of the heat wave season each year. The start of the first heat wave was counted as the number of days that had elapsed from 1 January. The same process was followed to count the end of the last heat wave. This process provided information on whether the heat wave season started earlier or lasted longer or both for a particular year.

The average yearly proportion of a newly enlarged urban area to the total area is the urban expansion intensity index (UEII) for a spatial unit [38]. Urban expansion can occur in different directions in every urban area depending on its driving factors, including road networks, population density, slope, economy, etc. [35]. UEII provides a quantitative assessment of urban spatial expansion. UEII can be expressed as follows:

$$UEII_{i}=\frac{ULA_{i}^{t_{2}}-ULA_{i}^{t_{1}}}{TLA_{i}\times \Delta t}\times 100$$

Here, $UEII_{i}$ is the urban expansion intensity index of the spatial unit i, $ULA_{i}^{t_{2}}$ is the area of the urban area at $t_{2}$ time, $ULA_{i}^{t_{1}}$ is the area of the urban area at $t_{1}$ time, $TLA_{i}$ is the total area of spatial unit i, and ∆*t* is the length of the study period.

GEII is used to measure the expansion or declination of green areas in a geographic boundary each year. It uses the same metric as the UEII, the only difference is it uses the green areas instead of the urban areas. In this study, the green area has been classified by the aggregation of particular classes of NLCD 1992 and NLCD 2019. The green expansion intensity index denotes the growth of a spatial unit’s green areas as a percentage of the overall area of the land unit during the study period and describes the degree of differentiation of green expansion in different directions. GEII can be expressed as follows:

$$GEII_{i}=\frac{GLA_{i}^{t_{2}}-GLA_{i}^{t_{1}}}{TLA_{i}\times \Delta t}\times 100$$

Here, $GEII_{i}$ is the urban green expansion intensity index of the spatial unit i, $GLA_{i}^{t_{2}}$ is the area of the green area at $t_{2}$ time, $GLA_{i}^{t_{1}}$ is the area of the green area at $t_{1}$ time, $TLA_{i}$ is the total area of the spatial unit i, and Δt is the length of the study period.

Spearman correlation transforms the data from its original scale towards a common scale, which is usually a rank. The Spearman coefficient is an indicator to determine the relationship of the bivariate distribution. This can be thought of as a measure of the underlying relationship’s monotonicity. A monotonic relationship does one or more of the following things: (1) as one variable value rises, so does the value of the other variable; (2) as one variable value rises, the value of the other variable decreases. The value of coefficients is characterized by the study by Fowler et al. [43] (Table 3).

## 3. Results

### 3.1. Urban Expansion Intensity Index

In this study, the UEII was determined for 27 years. Al-Sharif et al. [35] divided the values of UEII into certain classes. The descriptive statistics (Table 4) and the classification table (Table 5) show that the overall expansion rate of urban areas in the U.S. (mean 1.5%) can be classified as high-speed development every year. The top-five cities with the highest UEII values fall in the category of very high-speed development (Table 6). In terms of the UEII, the city of St. Louis in Missouri ranks first. The other four top cities (Chicago, Hartford, Detroit, and Milwaukee) have almost the same level of urban expansion rate (in percentage) each year. In contrast to the high- to very-high-speed development, New Orleans in Louisiana has developed its urban areas with an average value of 0.2% every year. This might be the strong influence of Ian McHarg’s “Design with Nature” on New Orleans’ urban design and planning [44]. Additionally, Jacksonville, San Francisco, and Norfolk fall into the low-speed development (Table 6) category, whereas the urban expansion rate of Nashville in Tennessee can be classified as medium-speed development, with a value of 0.8%.

### 3.2. Green Expansion Intensity Index

There are links between the frequency of extreme heat events and the loss of regional vegetative cover [45]. This study aimed to quantify the correlation by first calculating the GEII for 49 cities across the United States. The descriptive statistics are shown for the 49 U.S. cities, as well as mentioning cities with the highest and lowest GEII values (Table 7 and Table 8).

### 3.3. Heat Waves and Spearman Correlation Analyses

A study by Gubernot et al. [46] analyzed the heat-related fatality rates from 2000 to 2010. They showed that the rates in Florida, Texas, and California were 0.37, 0.33, and 0.24 per million workers per year, respectively, which are listed among the top ten states. Between 2000 and 2010, Texas and California accounted for nearly a quarter of all heat-related deaths. The highest changes were also seen in the same states, as seen in Table 9.

The Spearman correlation uses the UEII and GEII as dependent variables and frequency change, duration change, season change, and intensity change as the independent variables. The correlation coefficients show the relationship between the variables. The correlation has been done for the whole dataset and within the regions individually (Table 10, Table 11, Table 12 and Table 13). Statistically significant correlations were found between the UEII and duration change, and UEII and intensity change, based on the overall dataset of the U.S. The UEII and intensity change are statistically correlated in the West region.

## 4. Discussion

The UEII method was used to analyze the characteristics of land use morphology [38,47,48]. Shenghe et al. [47] analyzed the urban expansion speed of Beijing in China by using UEII. Hwang et al. [48] used the UEII method to examine the changing urban spatial expansion in the city of Seoul and its vicinity areas in South Korea. They reported that the expansion intensity index of Seoul ranged from 1.95 to 9.13 for the years 1975 and 1995, indicating a very rapid urban spatial expansion. Kang et al. [38] explored urban changes in the Democratic People’s Republic of Korea by using UEII. They calculated the UEII for two 10-year intervals, and the spatial autocorrelation of UEII values was examined for the entirety of North Korea.

The comparison between GEII and UEII clearly shows that the green area is not increasing at the same rate as urban expansion. The mean GEII of 0.017% and the mean UEII of 1.5% indicate that the urban expansion is more evident than green expansion in the 49 cities of the U.S. as an annual average. Maimaitijiang et al. [49] studied the St. Louis metropolitan and observed that the rate of buildup of land expansion was 58.99%, which was 6.1 times faster than the rate of population increase (9.74%). The reason for the rapid urban expansion was assumed to be a social preference for low-density housing, a suburban lifestyle, and a well-developed highway system. In this case, the population increase did not play such a rapid role as it was seen in Xuzhou city [36], because the population in St. Louis declined from 1970 to 2010 by 48.7%. Green areas in the two cities (Tucson and Phoenix) have been increasing from 1992 to 2019 by 1.45 and 1.13%, respectively, as seen in the annual average. Charlotte has the lowest GEII value of −0.9% and a UEII value of 1.62%. The high-speed development might be related to maintaining green areas in the cities. Milwaukee and St Louis have a GEII of −0.2% with a very high value of UEII (2.5 and 3.1% respectively). It will be beneficial to investigate the land use policies of the two cities for a better understanding of the relations between urban and green areas.

Spearman correlation analysis within the 49 cities in the U.S. revealed that the heat wave duration and intensity changes are significantly linked to UEII. Salvati et al. [50] used the correlation method for the evaluation of urban landscape and forest conservation in the outskirts of the city of Rome, Italy. Zambon et al. [51] analyzed multi-core urban developments and their economic growth in Europe using the Spearman analysis method. In this study, duration change was negatively correlated with UEII. It means that the cities expanding every year more rapidly might receive fewer days of the consecutive heat wave. However, UEII was positively and significantly (p = 0.02699) related to heat wave intensity, with the Spearman correlation coefficient of 0.317. These results might be interpreted as follows; First, there is a negative relation between heat wave duration and intensity from the perspective of an urban expansion intensity. Second, heat wave intensity can be a public health issue in high urban expansion intensity areas. A previous study suggests that urban green space decreases the intensity of the heat wave [24]. However, the relationships between GEII and heat wave frequency, duration, season, and intensity were not statistically significant in this study. The green spaces can be further analyzed in this instance. According to Xiao, X. D. et al. [52], large green spaces had a more noticeable and consistent cooling and humidifying effect, but small green spaces had a more variable cooling effect, with a heat preservation phenomenon happening in some circumstances. Also, another study [53] explained that the extent and timing of localized cooling effectiveness, particularly during high-intensity heat waves, is uncertain. Further emphasis on the green space type and shape might give a better explanation for the weak correlation.

The regional effect was negligible in this study. The heatwave characteristics have little correlation to the UEII and GEII regionwide which demands further investigation.

**Figure 3:**
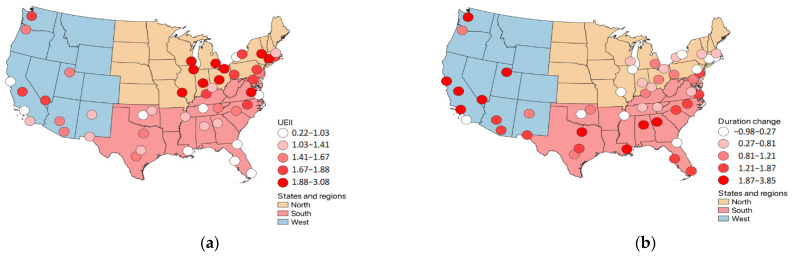
The relationship between UEII and duration change values; (**a**) spatial pattern of UEII values; (**b**) spatial pattern of duration change values.

**Figure 4:**
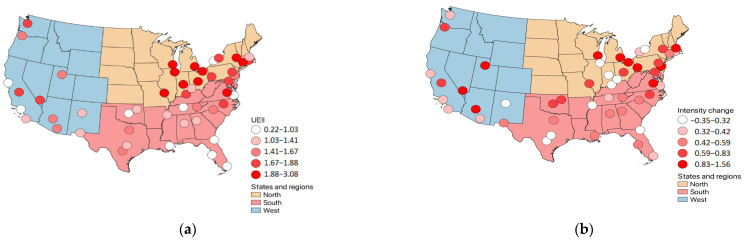
The relationship between UEII and intensity change values; (**a**) spatial pattern of UEII values; (**b**) spatial pattern of intensity change values.

Figure 3 and Figure 4 show the results graphically. Figure 3 shows that there is a negative relation between UEII and duration change values. This can also be verified in Table 3 and Table 5. Chicago and St. Louis have UEII values of 3.1 and 2.5, respectively, which can be considered as being very high-speed developments in the urban areas [35]. Figure 4 shows a similar pattern between UEII and intensity change values. The pattern shows that the value changes are in the positive direction. Philadelphia, Pittsburgh, Salt Lake, Boston, and Cleveland are the top five cities in intensity change (Table 3). The UEII values for these cities range from 1.1–2.2, where only one value (for Boston) falls below the mean UEII value of 1.5. The regions specifically do not exhibit strong correlations. The West region shows a significant and positive correlation only between UEII and intensity change. The coefficient value is 0.64 and the *p*-value is 0.03. Thus, the higher the UEII value, the higher the intensity of the heat wave. Seattle, Las Vegas, and Fresno have the highest UEII values among the 12 cities of the West region, which are 1.78, 1.75, and 1.71, respectively. The intensity changes for those cities are above the average of 0.62, except for Seattle (0.42).

## 5. Conclusions

Cities are increasingly concerned with new difficulties including the detrimental effects of heat waves, as the effects of climate change continue to grow. Further empirical research on urban and green spaces could give urban planners feasible implications for this issue. This paper presents the urban and green area changes using UEII and GEII. The relations of UEII and GEII were examined for the heat wave characteristics including frequency, season, duration, and intensity. It was found that heat wave intensity can be a public health issue in high urban expansion intensity areas. The results imply that the cities would be better in a more compact pattern with more expanded green areas to mitigate the negative health impacts of heat waves on citizens in urban areas. It is noticeable that there are some patterns to be investigated further in the context of urban developments and heat wave characteristics. Though this study has important implications for the public health issue of urban areas, it will be beneficial to use geographically heterogeneous data and sophisticated statistical methodology for an in-depth discussion regarding urban heat wave issues. The land use classification process can be performed by using remote sensing. It can be further cross-referenced with field data. Spearman’s correlation method allows the monotonic association between two variables. Different analytical approaches will be required for us to understand dynamic relations between land uses and heat wave characteristics.

## Figures and Tables

**Table 1:** Urban area classes for 1992 and 2019 NLCD.

Urban Area			
NLCD 1992 Classes		NLCD 2019 Classes	
Code	Classification Name	Code	Classification Name
21	Low Intensity Residential	21	Developed, Open Space
22	High Intensity Residential	22	Developed, Low Density
23	Commercial/Industrial/Transportation	23	Developed, Medium Density
		24	Developed, High Density

**Table 2:** Green area classes for 1992 and 2019 NLCD.

Green Area			
NLCD 1992 Classes		NLCD 2019 Classes	
Code	Classification Name	Code	Classification Name
41	Deciduous Forest	41	Deciduous Forest
42	Evergreen Forest	42	Evergreen Forest
43	Mixed Forest	43	Mixed Forest
51	Shrubland	51	Dwarf Shrub
		52	Shrub
71	Grasslands/Herbaceous	71	Grassland/Herbaceous
		72	Sedge/Herbaceous
61	Orchards/Vineyards/Other	73	Lichens
62	LULC Orchards/Vineyards/Other	74	Moss

**Table 3:** Spearman correlation coefficient value and interpretation.

$\mathrm{Value}\;\mathrm{of}\;\mathrm{Coefficient}\;r_{s}\;(\mathrm{Positive}\;\mathrm{or}\;\mathrm{Negative})$	Meaning
0.00–0.19	A very weak correlation
0.20–0.39	A weak correlation
0.40–0.69	A moderate correlation
0.70–0.89	A strong correlation
0.90–1.00	A very strong correlation

This study used the value of coefficients to determine the relation between the characteristics of heatwaves and UEII and GEII.

**Table 4:** Descriptive statistics of UEII for 1992–2019 for 49 U.S. cities.

Feature	UEII Values (in Percentage)
Mean	1.5
Median	1.53
Standard Deviation	0.61
Range	2.88
Minimum	0.22
Maximum	3.1

**Table 5:** UEII range and class.

UEII Range (in Percentage)	Class
0.00–0.28	Slow Development
0.28–0.59	Low-Speed Development
0.59–1.05	Medium-Speed Development
1.05–1.92	High-Speed Development
>1.92	Very High-Speed Development

Source: Al-Sharif, Pradhan, Shafri, and Mansor (2014) [35].

**Table 6:** Top and bottom five U.S. cities with the highest and lowest UEII values.

	City Name	UEII Values (in Percentage)
U.S. Cities with Highest UEII Values	St. Louis, MO	3.1
	Chicago, IL	2.5
	Hartford, CT	2.5
	Detroit, MI	2.5
	Milwaukee, WI	2.5
U.S. Cities with Lowest UEII Values	New Orleans, LA	0.2
	Jacksonville, FL	0.4
	San Francisco, CA	0.5
	Norfolk, VA	0.5
	Nashville, TN	0.8

**Table 7:** Descriptive statistics of GEII for 1992–2019 for 49 U.S. cities.

Feature	GEII Values (in Percentage)
Mean	0.017
Median	−0.1
Standard Deviation	0.43
Range	2.35
Minimum	−0.9
Maximum	1.45

**Table 8:** Top and bottom five U.S. cities with the highest and lowest GEII values.

	City Name	GEII Values (in Percentage)
U.S. Cities with Highest GEII Values	Tucson, AZ	1.45
	Phoenix, AZ	1.13
	Albuquerque, NM	0.77
	Covington, KY	0.75
	El Paso, TX	0.65
U.S. Cities with Lowest GEII Values	Charlotte, NC	−0.9
	Knoxville, TN	−0.7
	Raleigh, NC	−0.5
	Atlanta, GA	−0.5
	Portland, OR	−0.5

**Table 9:** U.S. cities with the highest and lowest frequency, duration, season, and intensity change values.

	Frequency Change	Duration Change	Season Change	Intensity Change
U.S. Cities with Highest Change Values	New Orleans, LA	Fort Worth, TX	San Francisco, CA	Philadelphia, PA
	Tampa, FL	New Orleans, LA	New Orleans, LA	Pittsburgh, PA
	Miami, FL	Salt Lake City, UT	Tampa, FL	Salt Lake City, UT
	Austin, TX	San Francisco, CA	Atlanta, GA	Boston, MA
	San Francisco, CA	Los Angeles, CA	Miami, FL	Cleveland, OH
U.S. Cities with Lowest Change Values	Hartford, CT	Chicago, IL	Jacksonville, FL	San Antonio, TX
	Los Angeles, CA	San Diego, CA	Indianapolis, IN	Indianapolis, IN
	San Diego, CA	St. Louis, MO	Buffalo, NY	Rochester, NY
	Rochester, NY	Rochester, NY	San Diego, CA	Memphis, TN
	Jacksonville, FL	Memphis, TN	Rochester, NY	Chicago, IL

**Table 10:** Spearman correlation between UEII, GEII, and heatwave frequency, duration, season, and intensity for 49 cities in the U.S.

Independent Variable, *x*	Dependent Variable, *y*	*p*-Value	$r_{s}$-Value
GEII	Frequency Change	0.5485	0.0878106
GEII	Duration Change	0.5717	0.08255102
GEII	Season Change	0.4877	−0.1012245
GEII	Intensity Change	0.7397	−0.04857143
UEII	Frequency Change	0.3794	0.1283739
UEII	Duration Change	0.03916 *	−0.2962245 *
UEII	Season Change	0.5069	−0.09683673
UEII	Intensity Change	0.02699 *	0.3168367 *

Note: * *p* < 0.05.

**Table 11:** Spearman correlation between UEII, GEII, and heatwave frequency, duration, season, and intensity for 15 cities in North Region.

Independent Variable, *x*	Dependent Variable, *y*	*p*-Value	$r_{s}$-Value
GEII	Frequency Change	0.8025	−0.07142857
GEII	Duration Change	0.2316	0.3285714
GEII	Season Change	0.2212	−0.3357143
GEII	Intensity Change	0.8224	−0.06428571
UEII	Frequency Change	0.2479	0.3178571
UEII	Duration Change	0.4578	−0.2071429
UEII	Season Change	0.237	0.325
UEII	Intensity Change	0.3138	−0.2785714

**Table 12:** Spearman correlation between UEII, GEII, and heatwave frequency, duration, season, and intensity for 22 cities in the South Region.

Independent Variable, *x*	Dependent Variable, *y*	*p*-Value	$r_{s}$-Value
GEII	Frequency Change	0.3194	−0.2219085
GEII	Duration Change	0.5112	−0.1473744
GEII	Season Change	0.267	−0.2467532
GEII	Intensity Change	0.7054	−0.08526256
UEII	Frequency Change	0.6942	−0.08865048
UEII	Duration Change	0.6942	0.08865048
UEII	Season Change	0.7509	−0.0717109
UEII	Intensity Change	0.2784	0.2411067

**Table 13:** Spearman correlation between UEII, GEII, and heatwave frequency, duration, season, and intensity for 12 cities in the West Region.

Independent Variable, *x*	Dependent Variable, *y*	*p*-Value	$r_{s}$-Value
GEII	Frequency Change	0.1542	0.4405594
GEII	Duration Change	0.5578	0.1888112
GEII	Season Change	0.8344	−0.06993007
GEII	Intensity Change	0.9737	−0.01398601
UEII	Frequency Change	0.4709	0.2307692
UEII	Duration Change	0.4709	0.2307692
UEII	Season Change	0.256	0.3566434
UEII	Intensity Change	0.03011 *	0.6363636 *

Note: * *p* < 0.05.

## Data Availability

Some or all data and models that support the findings of this study are available from the corresponding author upon reasonable request.
